# Editorial: Advances in non-union diagnostics, therapy and the understanding of its pathogenesis: current concepts from bench to bedside

**DOI:** 10.3389/fsurg.2023.1287251

**Published:** 2023-09-11

**Authors:** Patrick Haubruck, Michael C. Tanner, Lars Helbig

**Affiliations:** ^1^ HTRG—Heidelberg Trauma Research Group, Clinic for Trauma and Reconstructive Surgery, Center for Orthopedics, Trauma Surgery and Spinal Cord Injury, Heidelberg University Hospital, Heidelberg, Germany; ^2^ Raymond Purves Bone and Joint Research Laboratory, Kolling Institute, Institute of Bone and Joint Research, Faculty of Medicine and Health University of Sydney, Royal North Shore Hospital, St. Leonards, NSW, Australia

**Keywords:** non-union, bone healing, bone regeneration, bone biology, bone reconstruction, osteoimmunology

**Editorial on the Research Topic** Advances in non-union diagnostics, therapy and the understanding of its pathogenesis: current concepts from bench to bedside

## Introduction

Despite years of dedicated research and advances across a variety of associated medical fields failed fracture healing resulting in non-union remains a frequent and persistently challenging clinical problem (1). Treatment of non-unions remains highly demanding and costly due to the associated impaired local osseous microbiology, associated bony defects as well as the high levels of infections. Studies investigating the factors contributing to the development of non-unions shed light on the complex and intricate nature of its pathogenesis and over the years led to the introduction of enhanced treatment modalities to improve the outcome of non-union treatment (2). Despite these recent advances an ongoing need remains to improve the understanding of the underlying osseous biology as well as cellular and molecular interactions within the broader osseous network to address persisting short-comings and limitations in current treatment and diagnostic modalities. Although over decades inflammation has been seen as a major disruptor to physiological bone healing (3) only recently the concept of osteoimmunology and a distinct codependency between osseous cells and immune cells has been established (4). The concept of osteoimmunology has the potential of creating a paradigm shift in the way we treat failed fracture healing and contributes to provide a more holistic understanding of the biology underlying bone fracture healing. This special topic provides an exciting overview over articles exploring the concept of osteoimmunology in concept with fracture healing and further ventures into evaluating novel diagnostics used to improve intraoperative decision making during surgical treatment of non-unions.

## Osteoimmunology and its role in failed fracture healing

Although the inflammatory response marks the beginning of the fracture healing cascade (5) an unrestrained inflammation is known to lead to disaster. Thus, a highly organized and spatiotemporal interplay between the osseous and immune cells seems to be crucial for successful bone healing (4). Interferon-gamma (IFN-y) signaling has been established as a key player orchestrating many immunological functions both part of the innate and adaptive immune system (Tang et al.). Interestingly, despite its pro-inflammatory function on immune cells, recent studies showed that IFN-y signaling serves an anabolic function when focusing on osseous biology (Tang et al.). In particular, IFN-y signalling leads to a osteoblastic activation via Runx2 and further upregulation of osteogenic factors (e.g., osterix, Alp, and osteocalcin) (Tang et al.). On the other hand, IFN-y inhibits osteoclastogenesis by down regulating c-Fms in monocyte-derived osteoclast precursors to reduce RANK and the subsequent RANK-induced osteoclast differentiation (Tang et al.). When focusing on the direct effects of IFN-y on bone biology, studies showed that presence of IFN-y seems to be vital to maintain normal bone mass. However, in-vivo a dichotomous function for substituted IFN-y suggested an highly individual patient dependent manner of IFN-y effects, which is further corroborated by studies showing that IFN-y gamma treatment is effective in preventing bone loss during rheumatoid arthritis as well as during estrogen deficiency (Tang et al.). Taken together this review highlights the importance of INF-y signaling on bone biology and how its understanding has direct implications to preserve bone mass and how to potentially counteract changes during pathological conditions. Emerging technologies allowed researcher to take the findings made on a broader cellular or systemic level one step further towards a single cell level. A recent single cell RNA sequencing study confirmed the importance of osteoimmunology by revealing a comprehensive intercellular interaction landscape between local immune cells and osteoblastic lineage cells (OBCs) (Wang et al.). Amongst others, the authors found that crosstalk between immune cells and OBCs resulted in a ligand receptor reaction of Jagged1 and Notch2 leading to an enhanced BMP-2 induced osteoblastic differentiation. Hereby, the authors highlighted that despite lessons learned on a systemic or macro level single cell studies are important to further understand crosstalk between immune and osseous cells (Wang et al.). When focusing on musculoskeletal trauma a corroborating study indicated a potentially crucial role for a dysregulated systemic immune response resulting in poor outcomes following trauma (Vantucci et al.). In particular, myeloid-derived suppressor cells (MSDCs) were identified as a potential target in restoring systemic immune homeostasis. Furthermore, targeted depletion of those cells resulted in a normalization of bone healing further supporting the importance of osteoimmunology in the context if bone healing.

## Novel diagnostics facilitate intraoperative decision making in non-union treatment

Although a variety of materials (autograft, xenograft, allograft, alloplastic) exist that are suitable for bone grafting as part of non-union treatment, the most challenging graft requiring not only substantial expertise of the surgeon but also a highly advanced infrastructure remains vascularized bone grafts having a vascular pedicle (6). In scaphoid non-union surgery the likelihood of a successful treatment increases from 47% to 88% when choosing a vascularized bone graft over a non-vascularized one (Mulica et al.). However, previously no diagnostic modality existed that allowed immediate and reliable evaluation of the patency of the vascular pedicle. In the current research topic Mulica et al., presented compelling data regarding the benefits of using an indocyanine green fluorescence (ICG) angiography intraoperatively. The authors were able to reliably detect patency of the vascular pedicles in all patients and furthermore document sufficient perfusion of the bone graft itself and in some cases even of the cancellous bone (Mulica et al.). Clinically despite the complex nature of the included cases no additional treatment was to achieve bony union, and the authors concluded that ICG angiography is a promising diagnostic tool in treating complicated cases of recalcitrant scaphoid non-unions.

## Conclusion

Failed fracture healing remains a severe and debilitating complication of an already traumatic event in the life of affected patients. Although the underlying pathophysiological mechanisms remain complex ongoing research efforts provide data contributing towards a better understanding of the contributing factors both on a locally or systemically level. The current research topic highlights the importance of the immune system during bone healing and while studies over the years indicated that the immune system is neither friend no foe when it comes to bone healing moderation and modulation of immunological factors involved might help to make the immune system at least our lasting ally. Finally, while surgical techniques are improving intraoperative decision making is based on the experience of the surgeon but also on objective diagnostical tools that are readily available. The current topic introduces such a diagnostic modality and by publishing these results we hope to encourage surgeons to evaluate and potentially incorporate these diagnostical tools into their surgical and clinical routine.
